# Mechanisms of Antiulcer Effect of an Active Ingredient Group of Modified Xiao Chaihu Decoction

**DOI:** 10.1155/2018/5498698

**Published:** 2018-04-17

**Authors:** Wei Liu, Mengling Yang, Xuejian Chen, Li Li, Aijun Zhou, Shuhe Chen, Pengtao You, Yanwen Liu

**Affiliations:** ^1^Key Laboratory of Resources and Chemistry of Chinese Medicine, Hubei University of Chinese Medicine, 1 Huangjiahu Road West, Hongshan District, Wuhan, Hubei 430065, China; ^2^Post-Doctoral Research Center of Mayinglong Pharmaceutical Group Co., Ltd., 100 Zhoujiawan Road, Hongshan District, Wuhan, Hubei 430060, China; ^3^Wuhan Hospital of Traditional Chinese Medicine, 49 Lihuangpi Road, Jiangan District, Wuhan, Hubei 430000, China; ^4^Dongguan Hospital of Traditional Chinese Medicine, 22 Songshanhu Road, Dongcheng District, Dongguan, Guangdong 523000, China; ^5^Hubei Provincial Hospital of Traditional Chinese Medicine, 4 Huayuanshan Road, Wuchang District, Wuhan, Hubei 430000, China

## Abstract

The present study aimed to investigate the antiulcer activities and mechanisms of action of an active ingredient group (AIG) of Modified Xiao Chaihu Decoction (MXCD). The gastroprotective action of the AIG was studied in ethanol-induced, pylorus ligature-induced, and acetic acid-induced* in vivo *gastric ulcer models. The enzyme-linked immunoadsorbent assay (tumor necrosis factor-*α* (TNF-*α*), prostaglandin E_2 _(PGE_2_), and epidermal growth factor (EGF)), nitrate reductase assay (nitric oxide (NO)), western blot analysis (Bax, Bcl-2, cleaved-caspase-3, and cleaved-PARP (poly (ADP-Ribose) polymerase)), histological analysis (HE), and immunohistochemical analysis (HSP-70, p-AKT, and PCNA) were used to evaluate the anti-inflammatory, antiapoptotic, and healing properties of AIG. Numerous mechanisms are involved in the antiulcer activity of AIG, including the increase of PGE_2_, NO, and EGF content and a reduction in TNF-*α* levels. The upregulation of HSP-70, p-AKT, and PCNA seems to be directly linked to the healing effect of AIG. Bax, Bcl-2, cleaved-caspase-3, and cleaved-PARP also play a key role in this process. The AIG exerted gastroprotective effects by reducing antisecretory, anti-inflammatory, and antiapoptotic mechanisms. In addition, it promotes cell proliferation. Therefore, activation of the PI3K/AKT signaling pathway may play an important role in cell proliferation.

## 1. Introduction

Peptic ulcer disease embraces both gastric and duodenal ulcers [1] which occur due to an imbalance between the offensive (chloridric acid and pepsin) and defensive (mucus and bicarbonate) factors [2]. The gastric ulcer is one of the major gastrointestinal disorders with increasing incidence and prevalence globally [3]. Excessive drinking, smoking, nonsteroidal anti-inflammatory drugs (NSAIDs), and* Helicobacter pylori *all contribute to gastric ulcer [4], and the gastric ulcer is characterized by necrosis, secretion of inflammatory mediators, infiltration of neutrophils, and induction of oxidative [5].

Treatise on Febrile diseases includes hundreds of classic formulas written by Zhang Zhongjing, a well-known doctor in Chinese history, and Xiao Chaihu Decoction (XCD) is known as its representative. Experimental studies and clinical practices have proved that XCD is effective in the treatment of liver diseases because it can block the development of hepatitis to liver fibrosis [6] and further to liver cancer through various pathways [7]. Clinically XCD is also used to treat stomach disease [8]. XCD has been used for roughly 2000 years and made a great contribution to the prosperity of the Chinese nation. In addition, it has received high attention by domestic and foreign scholars, especially those of Japanese origin [9]. In Japan, XCD is also called* Shosaiko-to *(SHO); Matsuta et al. investigated the antiulcer properties of SHO and provided evidence that SHO possesses the capability of protecting the rat gastric mucosa and is able to inhibit gastric acid secretions [10]. The original prescription is often used in Japan, and the modification of the prescription is often used in China. Zhou Aijun, a chief physician of the Dongguan Hospital of Traditional Chinese Medicine in the Guangdong province of China, took XCD as basic prescription and added Coptidis rhizoma, Atractylodis macrocephalae rhizoma, and Poria to compose the so-called Modified Xiao Chaihu Decoction (MXCD). The preparation of MXCD was based on traditional Chinese medicine theory of abidance by triple pathogens (given time, given location, and given people) with appropriate therapies according to different time, different regions, and individual differences. MXCD has been used clinically to treat hundreds of gastric ulcer patients and its highly significant treatment effect is extremely welcomed.

According to the category of chemical components contained in each herb of MXCD and based on the clinical observations of MXCD when treating gastric ulcers, we extracted and separated MXCD into volatile oil, alkaloid, phenolic acid, saponin, polysaccharide, and other substance fractions. We then carried out bioactivity screenings by using anti-HP [11], anti-inflammation [12], and pharmacology experiments and identified volatile oil, alkaloid, phenolic, and saponin as the effective substances of the prescription. In an* in vivo* rat and mouse model, gastric ulcers were induced by using pylorus ligation, followed by administration of acetic acid and absolute ethanol. Subsequently, the substance fractions on pharmacodynamics were studied in gastric ulcers and all showed significant pharmacological activity. Based on previous studies, we combined the active substance fraction of MXCD and investigated the mechanism on antigastric ulcer. Our promising results lay the foundation for the development of a novel MXCD-based preparation of traditional Chinese medicine.

## 2. Materials and Methods

### 2.1. Drugs and Chemicals

In our study, we used the following drugs: ranitidine hydrochloride capsules (#616035008) from Shandong rossing pharmaceutical group Co., Ltd. (Shandong, China); ELISA assay kits for PGE_2_, TNF-*α*, and EGF purchased from Multi Sciences Co., Ltd. (Zhejiang, China); assay kit for pepsin and nitric oxide (NO) purchased from Nanjing Jincheng Bioengineering Institute (Jiangsu, China). All chemicals used in buffers and other solutions were of analytical grade.

### 2.2. Animals

Kunming mice weighing 18–22 g (6-7 weeks old, 50% male and 50% female) and Wistar rats weighing 200–220 g (6-7 weeks old, 50% male and 50% female) were from The Center for Disease Prevention and Control (Hubei province, China (reg. no. SCXK (Hubei) 2015-0018)). Animals were housed at 22 ± 2°C under a 12-h light/12-h dark cycle and had access to food and water ad libitum. Animals were acclimatized and habituated to the new environment for at least a week before they underwent testing and were only used once during the study. The study was carried out following the “Principles of Laboratory Animal Care” [13].

### 2.3. Preparation of Test Samples

Preparation of active ingredient AIG solution was as follows: Bupleuri radix (dry root from* Bupleurum chinense DC.*), Zingiberis rhizome (dry root from* Zingiber officinale *Rosc.), and Atractylodis macrocephalae rhizome (dry root from* Atractylodes macrocephala *Koidz.) were mixed (15 : 16 : 10 ratio) using steam distillation to obtain the essential oil (named Part 1, yield: 0.8 mL/100 g of powdered material). Scutellariae radix (dry root from* Scutellaria baicalensis *Georgi), Pinelliae rhizoma praeparatum (dry root from* Pinellia ternata *(Thunb.)), Codonopsis radix (dry root from* Codonopsis pilosula *(Franch.)), Glycyrrhizae radix et rhizoma praeparata cum melle (dry root from* Glycyrrhiza uralensis *Fisch.), Jujubae fructus (dry fruits from* Ziziphus jujuba *Mill.), Coptidis rhizoma (dry rhizome from* Coptis chinensis *Franch.), Poria (dry sclerotium from* Poria cocos *(Schw.)), which were all identified by professor Chen (Hubei University of Chinese Medicine), were mixed (10 : 10 : 10 : 6 : 30 : 3 : 15 ratio). The solution was extracted with water and a combination of water decoction. The extract was condensed by using alcohol precipitation (70%), the filtrate, that did not have an alcohol taste, was pH adjusted with NaOH (1 mol/L) to pH = 10 and pH = 11, and was extracted using chloroform. Subsequently, the chloroform-extracted fraction was condensed and dried to obtain Powder 2 (yield: 1.2 g/100 g of powdered material). The pH value of the aqueous solution after the chloroform extraction was adjusted with HCl (1 mol/L) to pH = 3-4 and extracted by ethyl acetate. The fraction extracted by ethyl acetate was condensed and dried to obtain Powder 3 (yield: 0.65 g/100 g of powdered material). The pH value of the aqueous solution after the ethyl acetate extraction was adjusted with NaOH (1 mol/L) to pH = 7 and extracted by water-saturated butanol, and then the fraction extracted by water-saturated butanol was condensed and dried to obtain Powder 4 (the yield was 2.4 g/100 g of powdered material). Part 1, Powder 2, Powder 3, and Powder 4 (now called AIG) were mixed (ratio: 0.8 : 1.2 : 0.65 : 2.4), dissolved in water containing 0.5% Tween 80, and diluted to a concentration of 1.5 and 1.0 g powdered material per ml, which represents the high concentration for mouse and rats, respectively. The concentration solution was diluted to 0.75 and 0.5 g powdered material per ml to obtain the low concentration solutions for mouse and rats, respectively.

### 2.4. Preliminary Chemical AIG Assays

AIG is a mixture that consists of four main components: Part 1, Powder 2, Powder 3, and Powder 4. The HPLC method [14] was used to determine the content of 6-gingerol in Part 1, the berberine content in Powder 2 [15], and the baicalin content in Powder 3 [16]. In Powder 4, the total saponin content was determined by a spectrophotometric approach at a wavelength of 603 nm and using saikosaponin-a as a control.

### 2.5. Gastroprotection Activity

#### 2.5.1. Ethanol-Induced Gastric Ulcer

Mice were randomly divided into four groups of eight animals per group. Mice in the control group received sterile saline (10 mL/kg). The treatment groups received ranitidine (40 mg/kg) or different doses of AIG (1.5 and 0.75 g/kg) dissolved in water, for a period of seven days. The AIG dosage was chosen based on clinically used dosage and the low dose of mice (0.75 g/kg) equivalent clinical dosage of human. Mice were fasted for 24 h but had free access to drinking water prior to receiving an oral dose of saline (10 mL/kg), ranitidine (40 mg/kg), or AIG (1.5 and 0.75 g/kg). After 60 min, mice in all groups received 1 mL of absolute ethanol orally for the induction of gastric ulcers. One hour later, animals were euthanized and their stomachs excised. Each stomach was incised along the greater curvature and rinsed with cold saline to remove gastric contents and blood clots. The lengths of the lesions were measured using a vernier caliper [17]. The percentage of ulcer inhibition was calculated using the following formula: [(ulcer length_(control)_  − ulcer length_(treated)_)/ulcer length_(control)_] × 100% [18].

#### 2.5.2. Gastric Acid Secretion

Rats were divided into groups (*n* = 8 per group). After 24 h of fasting, rats were anesthetized, the abdomen was incised, and the pylorus was ligated. Immediately after the pylorus ligation, rats were treated with saline (10 mL/kg), ranitidine (30 mg/kg), or AIG (1.0 and 0.5 g/kg). Rats in the control group received 1 mL of sterile saline. Six hours after treatment, rats were euthanized by cervical dislocation, the abdomen was opened, and another ligature was placed around the esophagus in close proximity to the diaphragm. The stomachs were removed, and the gastric content was collected and drained into a graduated centrifuge. To determine the gastric juice volume, total acidity, and pepsin activity, gastric content was centrifuged at 448 g for 15 min at 4°C [19]. The total acidity was determined by titration with 0.01 N NaOH [20]. Pepsin activity was determined using assay kits according to the manufacturer's guidelines.

### 2.6. Healing Properties

#### 2.6.1. Acetic Acid-Induced Chronic Gastric Ulcer

Rats were randomly divided into four groups (*n* = 8 per group). After fasting for 24 h, rats were anaesthetized, the abdomen was exposed, and 0.05 mL of 30% acetic acid (v/v) was injected into the subserosal layer in the glandular part of the anterior wall to induced chronic gastric ulcers. The stomach was washed with saline to avoid adherence to the external surface of ulcerated region. The abdomen was then closed and animals were fed normally. On the second day after the ulcer induction, rats were treated with saline (10 mL/kg, control group), ranitidine (30 mg/kg, positive control), and AIG (1.0 and 0.5 g/kg). Rats were treated once a day by gavage for 14 consecutive days. Ranitidine was prepared in water immediately before administration. Rats were euthanized 24 h after the last administration, and stomachs were removed and opened via the great curvature. The lesion lengths were determined with a vernier caliper.

Each stomach was sectioned in half. One portion was fixed in 10% formalin for 24 h at 4°C, and the other portion of the stomachs was stored at −80°C for future biochemical analysis. These stomach samples were embedded in Paraplast, cut into 5 *μ*m sections, and routinely processed for histological evaluation.

#### 2.6.2. Cytokine Evaluation

Frozen stomach samples were homogenized in ice-cold saline using an Ultra Turrax Homogenizer (IKA, Germany) and centrifuged at 8,000*g* for 10 min at 4°C (Hitachi Koki, Japan). Cytokine levels of NO, PGE_2_, TNF-*α*, and EGF were determined in the supernatants by using commercially available enzyme-linked immunosorbent assay (ELISA) kits. An NO assay kit (nitrate reductase method) was used to determine NO levels. The absorbance was read at 550 nm for NO and 450 nm for PGE_2_, TNF-*α*, and EGF using a microplate spectrophotometer (Bio-Rad, USA)

#### 2.6.3. Expression Levels of Bax, Bcl-2, Cleaved-Caspase-3, and Cleaved-PARP

Frozen stomach samples from acetic acid-induced gastric ulcers were homogenized in 1.5 mL of ice-cold PBS. Homogenates were centrifuged (12,000*g*, 10 min, 4°C) and the protein concentration was determined by a BCA protein assay kit (Servicebio, China). Subsequently, samples were treated with Laemmli buffer (Tris-HCl buffer, glycerol, sodium dodecyl sulfate (SDS), Bromophenol, and *β*-mercaptoethanol) using a 1 : 1 ratio. Equal amounts of protein (100 *μ*g) were separated using SDS polyacrylamide gel electrophoresis on a 5% acrylamide gel. Proteins were then transferred onto a polyvinylidene fluoride (PVDF) membrane and incubated with specific antibodies: Bax (Servicebio, China), Bcl-2, cleaved-caspase-3, and cleaved-PRAP (Cell Signaling Technology, USA). Each membrane was washed three times for 5 min with TBST and incubated with anti-mouse/rabbit secondary antibody (Cell Signaling Technology, USA) at a 1 : 3000 dilution. Membranes were washed three times 5 min with TBST and membranes were evaluated for *β*-actin expression using an anti-*β*-actin mouse antibody (Cell Signaling Technology, USA). Immunodetection was performed using an ECL chemiluminescence reagent kit (Servicebio, China). Densitometric data were analyzed by using Alpha software program (Alpha Innotech, USA).

#### 2.6.4. Histological Analysis

Stomach samples from acetic acid-induced gastric ulcers were fixed in 10% formalin and embedded in paraffin. Paraffin sections, 5 *μ*m thickness, were cut and sections were stained with hematoxylin and eosin (H&E) for histological evaluation according to a standard approach [21].

#### 2.6.5. Immunohistochemical Analysis of HSP-70, p-AKT, and PCNA

For immunohistochemical studies, gastric tissue sections were dewaxed and dehydrated, and the antigen was retrieved by using the microwave (400 watt for 8 min, followed by 100 watt for 7 min). Endogenous peroxidase was blocked by incubation with a 3% hydrogen peroxide solution at room temperature for 25 min in the dark. Sections were then incubated with primary antibodies against PCNA (Servicebio, China) (1 : 500), HSP-70 (Ruiying Biological, China) (1 : 100), or p-AKT (Servicebio, China) (1 : 200). After rinsing in phosphate-buffered saline (PBS, 0.01 M/L, pH 7.4), sections were incubated with HRP-labeled goat anti-rat IgG (DAKO, Denmark) at room temperature for 50 min. Sections were then washed in PBS (5 min, 3 times) and incubated in chromogenic solution (3,3-diaminobenzidine tetrahydrochloride) (DAKO, Denmark). Nuclei were counterstained using hematoxylin. Densitometric data were analyzed by using Image-Pro Plus 6.0 software (Media Cybernetics, USA).

### 2.7. Statistical Analysis

All data are expressed as mean ± SEM and statistical analyses were performed using statistical product and service solutions (SPSS) software. The statistical significance of differences for each parameter among groups was analyzed using a one-way analysis of variance (ANOVA) followed by a Dunnett's test. A value of *P* < 0.05 was considered statistically significant.

## 3. Results

### 3.1. Gastroprotection Activity

#### 3.1.1. Ethanol-Induced Gastric Ulcers

**Figure 1:**
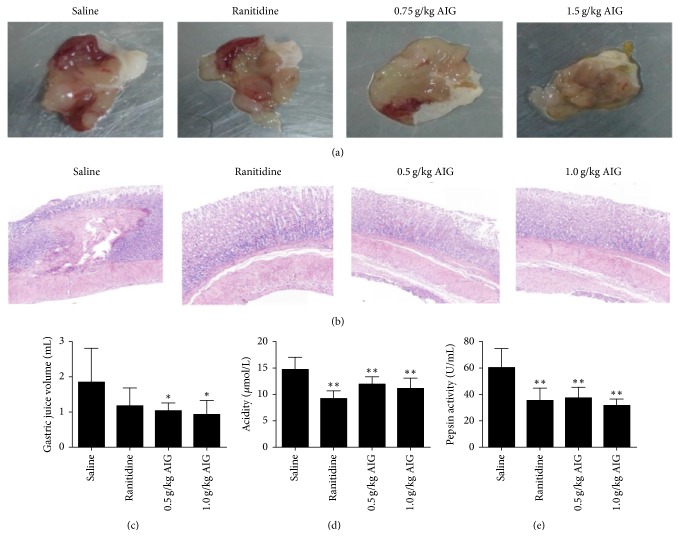
*Gastroprotective effects of AIG treatment on gastric ulcers induced by ethanol, pylorus ligature, or acetic acid.* Panel (a) shows representative macroscopic photographs of stomachs of gastric ulcers in mice induced by ethanol. Panel (b) represents histological analysis of gastric ulcers in rats induced by acetic acid (magnification 100x). Panels (c), (d), and (e) show the gastric juice volume, acidity, and pepsin activity in rats that underwent pylorus ligation. Gastric content was collected 6 h after ligation of the pylorus. Data are presented as the mean ± SEM and analyzed by one-way ANOVA followed by the Dunnett's test. ^*∗*^*P* < 0.05, ^*∗∗*^*P* < 0.01, compared to the saline-treated group.

Data showed that in the mouse model the ulcer control group presented severe mucosal injury. For ranitidine and AIG-treated groups, the ulcer area was significantly attenuated (*P* < 0.01). Ulcer index and inhibition are shown in Table 1, and ulcer areas are listed in Figure 1(a).

#### 3.1.2. Gastric Acid Secretion

Pretreatment with AIG significantly reduced gastric juice volume (*P* < 0.05) (Figure 1(c)) and acidity (*P* < 0.01) (Figure 1(d)). However, pepsin activity was significantly reduced after ranitidine treatment (*P* < 0.01) and AIG treatment (*P* < 0.01, Figure 1(e)).

#### 3.1.3. Acetic Acid-Induced Gastric Ulcer

Fourteen days after the induction of lesions, for ranitidine and AIG-treated groups, the ulcer area was significantly attenuated (*P* < 0.01) compared to control group (saline) (Table 1).

#### 3.1.4. Histological Analysis

Histological analyses of the gastric mucosa are depicted in Figure 1(b). In rats in the control group (saline treatment), severe damage was observed in the gastric epithelium. Rats pretreated with ranitidine (30 mg/kg) or AIG (0.5 g/kg) showed decreased mucosal damage when compared to the saline-treated group. However, rats pretreated with AIG (1.0 g/kg) showed normal histology. Only minor, superficial lesions were observed when compared to the saline-treated group.

### 3.2. Effect of AIG Treatment on NO, PGE_2_, TNF-*α*, and EGF Levels

**Figure 2:**
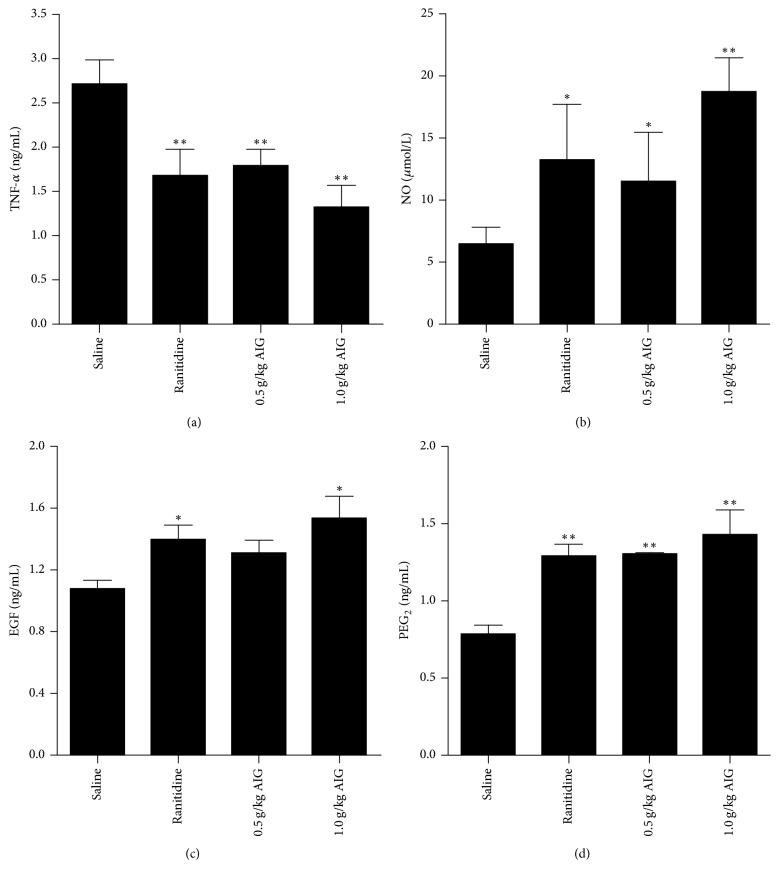
*Effect of ranitidine (30 mg/kg) and AIG (1.0 and 0.5 g/kg) on the levels of TNF-α (a), NO (b), EGF (c), and PGE*_2_ *(D) in gastric tissue in rats that were administered acetic acid.* Results are presented as mean ± SEM. Data were analyzed by one-way ANOVA followed by a Dunnett's test. ^*∗*^*P* < 0.05, ^*∗∗*^*P* < 0.01, compared to the saline-treated group.

In gastric tissue of rats, administration of AIG significantly reduced TNF-*α* levels (*P* < 0.01). Despite being treated with acetic acid, rats treated with AIG (1.0 g/kg) showed increased NO levels (*P* < 0.01) and maintained high levels of EGF (*P* < 0.05) and PGE_2_ (*P* < 0.01) in gastric tissue. Results are shown in Figure 2.

### 3.3. Expression of Bax, Bcl-2, Cleaved-Caspase-3, and Cleaved-PARP

**Figure 3:**
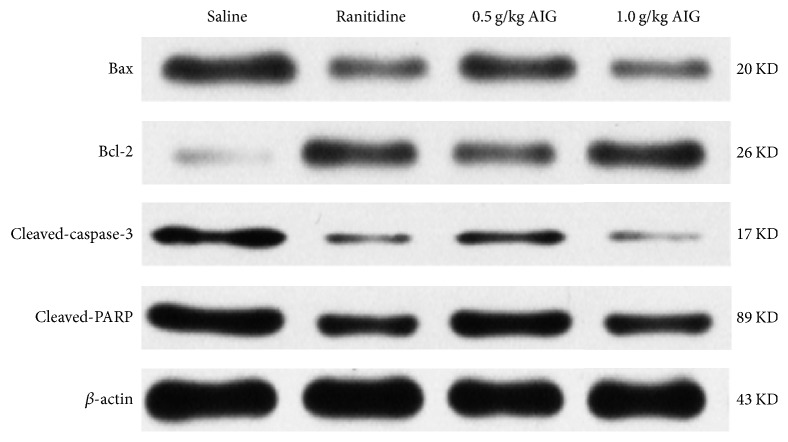
*Effect of ranitidine (30 mg/kg) and AIG (1.0 and 0.5 g/kg) on Bax, Bcl-2, cleaved-caspase-3, and cleaved-PARP expression in acetic acid-induced gastric tissue.* Proteins were analyzed by western blot analysis and expression was normalized to *β*-actin.

Western blot analysis revealed that when compared to the saline-treated control group, in both ranitidine and AIG-treated animals, Bcl-2 was upregulated and expression of Bax, cleaved-caspase-3, and cleaved-PARP expression was downregulated (Figure 3).

### 3.4. Immunohistochemical Analysis of HSP-70, p-Akt1, and PCNA

**Figure 4:**
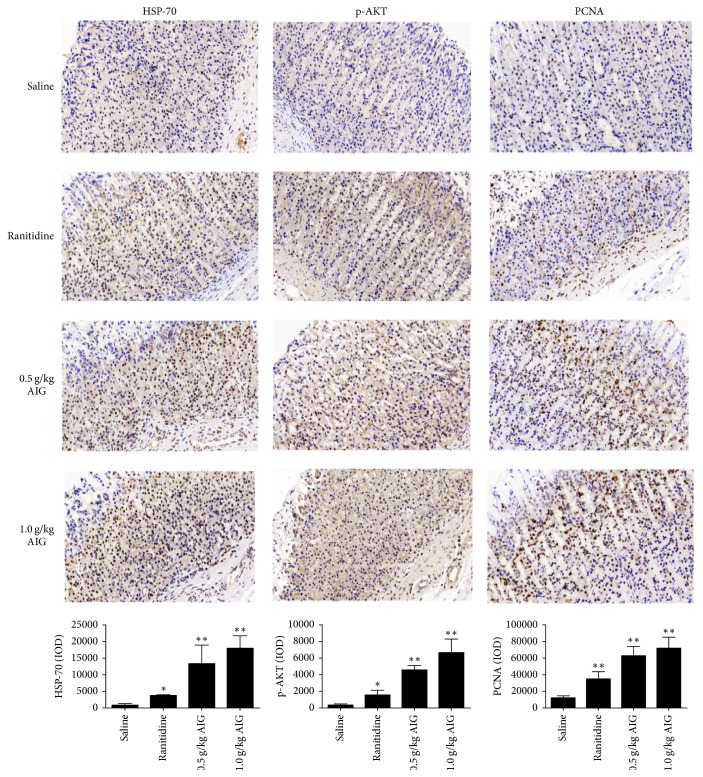
*Effect of ranitidine and AIG treatment on HSP-70, p-AKT, and PCNA expression in acetic acid-induced gastric tissue of rats.* Data are presented as mean ± SEM and analyzed by one-way ANOVA followed by Dunnett's test. ^*∗*^*P* < 0.05, ^*∗∗*^*P* < 0.01, compared to the saline-treated group. Original magnification: 400x.

Gastric tissues obtained from acetic acid-induced gastric ulcers were used for immunohistochemical analysis of HSP-70, p-AKT, and PCNA. Images were taken using a consistent background light, and then the optical density value (IOD) of the accumulated positive images was calculated. Analysis of histological sections demonstrated that, in stomachs of AIG-treated rats, HSP-70 levels were significantly increased when compared to saline-treated animals (*P* < 0.01). A similar increase was found for levels of p-AKT and PCNA (*P* < 0.01) (Figure 4).

## 4. Discussion

In modern medicine, it is well accepted that gastric ulcers are the result of a mucosal imbalance between attack and defense factors [22]. Matsuta et al. found that SHO possesses the capability of protecting the rat gastric mucosa as well as sucralfate and is able to inhibit gastric acid secretions like cimetidine or atropine, estimating that its mechanism might be related to the inhibition of attack factors and the activation of defense factors [10].

NO is an endogenous vasodilatation factor that can significantly expand gastric mucosa in blood vessels, improve blood flow to the stomach, maintain epithelial integrity of gastric mucosa, and promote mucosal repair after injury [23]. PGE_2_ is key for regulating gastric mucus secretion. PGE_2_ acts by improving blood flow to maintain cellular integrity in the mucosa and increases mucus secretion. In addition, PGE_2_ increases bicarbonate and sulfhydryl compounds to toughen the resistance of gastric mucosal cells to the necrotizing effect of strong irritants, such as ethanol, HCl, and acetic acid [24]. Previous studies have reported that PGE_2_ not only prevents the formation of irritant-induced gastric ulcers but also enhances the healing of gastric ulcers. For the effective treatment of gastric ulcers, both the prevention of further ulcer formation and the improvement of ulcer healing are important factors [25]. TNF-*α*, an inflammatory cytokine with pleiotropic activities, is actively involved in the process of inflammation. Extremely high levels of TNF-*α* are detected in the serum of rats with inflammatory diseases, such as in rats with severe mucosal inflammation. Moreover, the high TNF-*α* levels found in gastric lesion specimens obtained from gastric ulcer patients strongly support the hypothesis that TNF-*α* has detrimental effects, including the induction of tissue injury and inflammation [26]. As an inflammatory cytokine, TNF-*α* can cause a second release of other cytokines and activate neutrophils and endothelial cells that affect the mucosal blood oxygen supply, which may eventually lead to the formation of ulcers [27]. Our data showed that, after the administration of acetic acid, AIG treatment increased NO and PGE_2_ levels and decreased TNF-*α* levels (Figure 2). These results indicate that the increase in anti-inflammatory cytokine levels and decrease in proinflammatory cytokine levels may significantly contribute to the promotion of ulcer healing.

EGF is one of the most important factors that promote wound healing and have strong promotive mitotic activities on epithelial cells. EGF can promote the migration of epithelial cells, endothelial cells, and fibroblasts and, in addition, promotes angiogenesis and epithelial regeneration [28]. Therefore, EGF is considered to be a molecular regulation factor in the process of gastric mucosal injury [29]. In our study, we found an increase in the level of EGF (Figure 2(c)) in animals treated with AIG or ranitidine.

In normal gastric mucosa, there is a dynamic equilibrium between the process of damage and repair. The occurrence of cell apoptosis in gastric mucosa is closely related to ulcers [30]. Bax and Bcl-2 are important proapoptotic and antiapoptotic proteins, respectively. Bax and Bcl-2 are involved in regulating apoptosis [31]. When Bax expression upregulated, it can induce cell apoptosis by forming Bax/Bax dimers [32]. In addition, increased Bcl-2 expression inhibits apoptosis by forming Bcl-2/Bax dimers [33]. The caspase family also plays an important role in apoptosis. When an apoptosis signal is detected, caspases-9 are released from the mitochondria into the cytoplasm and the caspase cascade is activated after caspases-9 combining with Apaf-1. Caspase-3 plays a central role in the cell apoptosis and is responsible for the cleavage of PARP during cell death [34]. Our results demonstrated that AIG upregulates the expression of Bcl-2 and downregulates the expression of Bax and cleaved-caspase-3 (Figure 3). Moreover, cleavage and decreased expression of downstream PARP (Figure 3) inhibited apoptosis. Our results indicate that AIG inhibits apoptosis of gastric tissue cells, thereby promoting the healing of ulcers.

HSP-70 is considered one of the most important stress proteins [35]. When cells are exposed to gastric irritants, for example, ethanol [36], the expression of heat shock proteins is increased, which increases the resistance of the cells to the irritants [37]. Recently, Ishihara et al. investigated the role of HSP-70 in gastric ulcer healing and provided evidence that HSP-70 accelerates the process of healing by increasing the level of PGE_2_ [38] and expression of growth factors [39]. Moreover, the cell protective effect of HSP-70 is related to the activation of protein kinase C (PKC) and the inhibition of inflammatory mediator expression such as TNF-*α* [40]. Figures 2 and 4 demonstrate a significant increase in PGE_2_ and HSP-70 expression and a decrease in TNF-*α* levels in AIG-treated rats. Expression of HSP-70 increases the survivability of cells. Cells in which HSP-70 is knocked down are sensitive to apoptosis [41]. Overexpression of HSP-70 inhibits apoptosis and affects the expression of transcription factors that are associated with proteins of the Bcl-2 family [42] which act either downstream or upstream to mitochondria [43]. Furthermore, HSP-70 interacts with growth factors and enhances cell survival by activating the PI3K/AKT signaling pathway. Activated AKT initiates a growth factor-mediated survival signal and is involved in endothelial cell migration and tube formation [44]. In this context, the data generated in this study are in line with the data that show that HSP-70 accelerates the process of healing in gastric ulcer by activating the PI3K/AKT signaling pathway (Figure 4) and inhibits apoptosis (Figure 3).

As previously mentioned, cell proliferation plays a critical role in gastric ulcer healing and the PI3K/AKT signaling pathway is considered one of the most important regulating pathways of cell proliferation [45]. Factors that can activate the PI3K/AKT pathway include EGF, VEGF, and insulin. AKT is known to block apoptosis via phosphorylation of various downstream signaling molecules [46], including the activation of the Bcl-2 family member Bad and the inhibition of the cell death pathway enzyme caspase-9 [47]. Moreover, AKT activation can inhibit levels of the cell periodic dependence protein kinase inhibitor P21, thereby promoting the release of PCNA and promoting proliferation and survival of gastric mucosal epithelial cells. Quantitative immunohistochemical analysis demonstrated a significant increase of p-AKT and PCNA levels in acetic acid-induced gastric tissue of rats treated with AIG (Figure 4), indicating that AIG-associated reduction in gastric mucosal damage is primarily regulated via the AKT pathway.

## 5. Conclusion

The present study demonstrated that AIG treatment exerted protective effects in ethanol-induced, pylorus ligature-induced, and acetic acid-induced gastric ulcer models. These findings suggested that the mechanisms underlying the gastroprotection exhibited by AIG are antisecretory and lead to reduced secretion of proinflammatory mediators such TNF-*α* and elevated levels of anti-inflammatory cytokines including NO and PGE_2_, increased EGF activation, and upregulated expression of HSP-70, p-AKT, and PCNA. In addition, the Bax/Bcl-2 ratio, which leads to cell proliferation, was downregulated. In conclusion, elucidation of the underlying mechanisms of action helps put the traditional use of MXCD for the treatment of gastrointestinal diseases on a solid scientific footing.

## Figures and Tables

**Table 1:** *Effect of AIG treatment in ethanol-induced gastric ulcers in mice, pylorus ligature-induced, and acetic acid-induced gastric ulcer in rats.* Data are presented as mean ± SEM and analyzed by ANOVA followed by a Dunnett's test.

Gastric ulcer model	Treatments (p.o.)	Dose (g/kg)	*n*	Ulcer index (mm)	Inhibition (%)
Ethanol-induced	Saline	–	8	14.37 ± 2.88	–
	Ranitidine	0.04	8	11.27 ± 2.49^*∗∗*^	29.01
	AIG	1.50	8	3.11 ± 0.47^*∗∗*^	80.43
	AIG	0.75	8	4.46 ± 0.51^*∗∗*^	71.93
Pylorus ligature-induced	Saline	–	8	11.33 ± 3.78	–
	Ranitidine	0.03	8	3.55 ± 0.63^*∗∗*^	68.68
	AIG	1.0	8	2.76 ± 0.67^*∗∗*^	75.63
	AIG	0.5	8	3.36 ± 0.78^*∗∗*^	70.33
Acetic acid-induced	Saline	–	8	10.26 ± 1.52	–
	Ranitidine	0.03	8	3.89 ± 0.48^*∗∗*^	64.37
	AIG	1.0	8	2.84 ± 0.53^*∗∗*^	72.49
	AIG	0.5	8	3.38 ± 0.64^*∗∗*^	63.15

^*∗∗*^ *P* < 0.01, significantly different from saline-treated animals.
